# Establishment and characterization of Cri Du Chat neuronal stem cells: a novel promising resource to study the syndrome

**DOI:** 10.1007/s13577-025-01230-x

**Published:** 2025-05-09

**Authors:** Giovanna Piovani, Rosalba Monica Ferraro, Silvia Clara Giliani

**Affiliations:** 1 Department of Molecular and Translational Medicine, University of Brescia, 25123 Brescia, Italy; 2 Scientific Committee of A.B.C. Associazione Bambini Cri du Chat, 50026 Florence, Italy; 3 “A. Nocivelli“ Institute for Molecular Medicine, Department of Molecular and Translational Medicine, University of Brescia, 25123 Brescia, Italy

**Keywords:** IPSCs-NSC, Cri du Chat syndrome, Cri du Chat neurons, Deletion chromosome 5p, Disease modeling

## Abstract

The Cri Du Chat (CdC) Syndrome is a rare chromosome disease condition resulting from variable size deletion occurring on the short arm of one of the chromosomes 5. This disorder, which affects one in 50,000 births, is responsible for developmental retardation, the mechanism of which has remained unexplained. *TERT, SEMA5 A, CTNND2, TPPP,* mapped in chromosome 5 short arm, are known to be expressed in the brain, and to play a role in the development of the nervous system, oligodentrocytes and in the regulation of glutamatergic and dopaminergic synaptic transmission. It is critical to understand how their haploinsufficiency might affect the development and presentation of the disease. In the absence of an animal model and of significant accessible, human tissue, human pluripotent stem cells (iPSC) directly reprogrammed from patient somatic cells open a new area of disease modeling as they can virtually be differentiated into any cell type. Our study reports, for the first time, the generation of neuronal stem cells (NSCs) from CdC-iPSCs line and in addition, subsequent differentiation into a heterogeneous population of neurons. Gene expression of the mentioned and single copy deleted genes was also evaluated by comparing their expression level in iPSC, NSCs and neuron lines. The present research represents the first and the most innovative approach, to create an in vitro CdC neuronal model to have a new translational framework to study the pathologic processes.

## Introduction

Cri-du-Chat (CdC) syndrome is a rare genetic disease caused by total or partial deletion in the short arm of chromosome 5 [1] with the incidence of 1:50,000 live-born infants [2–4]. The main clinical features are a high-pitched monochromatic cry, microcephaly, severe psychomotor and mental retardation. Children have hyperactivity, with clinical features of attention deficit disorder [5], impulsiveness, temper tantrums, obsessive–compulsive disorder, poor concentration, aggressive behaviors, such as biting, hair pulling, pinching, and hitting, and self-injurious behaviors. Individuals may have characteristics of neurodegenerative and autism spectrum disorders [6]. The most frequent structural brain abnormalities in CdC patients, as shown by the few studies available in the literature using MRI, include brain stem hypoplasia, with predominantly pontine involvement, thinning or agenesis of the corpus callosum, cerebellar vermian atrophy or agenesis, cerebellar cortical thickening, and incomplete arborization of the white matter [7, 8]

Using 18 F-FDG PET/CT, the differences in brain glucose metabolism between CdC patients and control subjects were disclosed [9].

A study performed after autopsy revealed neuronal intracytoplasmatic inclusion positive for α-Synuclein, Tau, β-amyloid. The brain exhibited minimal inflammation without evidence of infection. Electron microscopy demonstrated Lewy-body (LB)-like inclusions in the neurons. These neuropathological features suggested α-Synucleinopathy with LB-like pathology in CdC disease [10].

Studies, performed in silico for deleted genes in CdC syndrome, highlighted the link between inflammation related to NF-kB/IL1 activation, TERT-induced TNF-a reduction and IL6-induced inflammation [11].

Loss of several genes in the 5p region contributes to the phenotype and to play a role in the development of the nervous system. In particular, the *CTNND2, SEMA5 A, TPPP, TERT* genes seem to be involved in the process of neuronal development and the deletion of one allele could contribute to the syndrome phenotype.

*CTNND2,* delta-catenin protein, is expressed early in neurons and is thought to play a role in neuronal migration. A mouse model showed that δ-catenin regulated the maintenance of dendrites and dendritic spines in mature cortex of the mouse. [12–15]. *SEMA5 A,* Semaphorin, is one of a large class of proteins that function throughout the nervous system to guide axons [16]. *TPPP*, TPPP/p25, is a microtubule associated protein, expressed in the human brain that modulates the dynamics and stability of the microtubule system, where is primarily engaged in the development of projections of oligodendrocytes that are responsible for the ensheathment of axons [17–19]. The non-physiological expression levels of this protein lead to distinct diseases such as synucleinopathies [20, 21].

*TERT* encodes for a ribonucleoprotein polymerase, that maintains telomere ends by addition of the telomere repeat TTAGGG [22]. Telomerase expression plays a role in cellular senescence. Studies in mouse suggest that telomerase also participates in chromosomal repair and has additional roles in cell survival, mitochondrial function, DNA repair, and Wnt signaling, all of which are unrelated to telomeres [23, 24]. *TERT* is enriched in Purkinje neurons, is expressed in the neonatal brain of mice as well as in distinct regions of the adult mouse brain such as the olfactory bulb, subventricular zone, hippocampus, cortex [25, 26]. Recently, many additional activities exhibited by TERT have been identified. This indicates that TERT may has telomere-independent biologic functions [27–29].

While it is clear that these genes located in the deleted region could play a crucial role for the development and function of the nervous system, nonetheless we are far from a conclusive agreement on their function and the consequences of their deletion in hemizygosis on the CdC syndrome.

To date, animal models are not available for CdCS study. Induced pluripotent stem cells (iPSCS) represent an accurate model to investigate the physiopathology of affected human cells and a suitable tool to test drugs or novel therapeutic approaches [30–36].

We have previously reprogrammed to pluripotency peripheral blood mononuclear cells (PBMCs) derived from CdC patient [37]. Starting from these cells, we have differentiated and characterized CdC-Neuronal Stem cells (CdC-NSCs) and in addition, subsequent differentiation into a heterogeneous population of neurons. Our research aims to perform an initial analysis, so far undocumented, on neuronal cells that could express important signs related to the Cri du Chat patient’s neurodevelopmental delay.

For this research we used healthy donor iPSCs, as a control, and the CdC-IPSCs lines so that we could compare results by highlighting discrepancies between normal and pathologic differentiated cells. We obtained the first very important results on a heterogeneous neuronal population differentiated from CdC-IPSCs and, for the first time, expression data of the mentionated CdC deleted genes involved in neuronal process.

## Material and methods

### hiPSC lines

We used healthy donor (HD) hiPSCs line, as a control, [38] and the UNI BSi004-A hiPSCs line derived from patient with CdC syndrome [37]. Reprogramming was previously described [37–39].

### Differentiation of CdC-iPSCs into CdC-neural stem cells (CdC-NSCs)

The neuronal differentiation process consisted of the initial neural induction of iPSCs, and than the differentiation of the different subtypes of neurons [40–43]

A protocol based on PSC Neural Induction Medium, containing Neurobasal medium and Gibco PSC neural induction supplement (Thermo Fisher Scientific, catalog number: A1647801), a serum-free medium able to induce efficiently differentiation of iPSCs into NSCs, without requiring intermediate step of embryoid body (EB) formation or co-cultures with stromal cell lines, thus reducing experimental variability and time consuming, has been utilized to produce NSCs [41, 44].

iPSCs were split as small cell clumps into Matrigel-coated 6-well plates at a density of 3 × 10^4^ cells per cm^2^. Twenty four h after splitting, culture medium was switched to PSC Neural Induction Medium. Neural induction medium was changed every other day from day 0 to day 4 of neural induction. After day 4 of neural induction, neural induction medium was changed every day as cells reached confluence. At day 7 of neural induction, passage 0 NSCs (NSCs p0) were dissociated with StemPro Accutase (Thermo Fisher Scientific, catalog number: A11105-01) and plated on Matrigel-coated 6-well plates at a density of 5 × 10^4^ cells per cm^2^ in an NSC expansion medium containing 50% Neurobasal medium (Thermo Fisher Scientific, catalog number: 21103049), 50% Advanced DMEM/F12 (Thermo Fisher Scientific, catalog number: 12634), and neural induction supplement. NSC expansion medium was changed every other day until NSCs reached confluence. For NSCs before passage 4, 5-μM ROCK inhibitor Y27632 (Sigma-Aldrich, catalog number: Y0503) was added to NSC expansion medium at the time of NSCs plating to treat cells overnight for the prevention of cell death. Dissociated NSCs were collected for cryopreservation, expansion, and characterization.

### Cytogenetic characterization of neuronal stem cells

NSCs at passage 6, undergoing active cell division, were blocked at metaphase by 10 μg/ml of colcemid (Karyo Max, Gibco Co. BRL), detached by trypsin–EDTA, and subsequently swollen by exposure to hypotonic KCL (0,075 M) solution. The cells were fixed with methanol/glacial acetic acid (3:1) three times and dropped onto glass slides. Cytogenetic analyses were performed using QFQ-banding at 450 bands resolution. A minimum of 20 metaphase spreads and 3 karyograms were analyzed [45].

To validate the deletion, Fluorescence in situ hybridization (FISH) analysis was carried out by standard procedures as described in the protocol provided by the probe manufacturer.

4′,6-Diamidino-2-Phenylindole, Dilactate (DAPI) staining was used for chromosome identification during FISH analysis. FISH results were analyzed using a Zeiss Axiophot microscope (Zeiss, Jena, Germany) with a triple bandpass filter to detect multiple signals simultaneously DAPI/FITC/Texas Red. Images were collected using a cooled CCD camera and subsequently merged. The probe used is CdCCR (Cri du Chat Critical Region) (5p15) green/(5q31) red, Kreatech; the green signal maps to the region of interest, while the red signal is the control signal (5qter).

### Neuronal differentiation of NSCs

Expanded NSCs at passage 10 were used for neuronal differentiation. Accutase-dissociated NSCs were plated onto poly-L-ornithine (Sigma-Aldrich, catalog number: P3655) and Laminin (ThermoFisher Scientific, catalog number: 23017015) coated 6 well plates in StemPro NSC SFM (Thermo Fisher Scientific, catalog number: A1050901) at 5 × 10^4^ cells/cm^2^ for 2 days. This medium allows the proliferation of neuronal precursors. After 2 days, the medium was switched to neural differentiation medium composed by Neurobasal Medium supplemented with B-27 Supplement serum free (50x, Thermo Fisher Scientific, catalog number:17504044), GlutaMAX-I Supplement (100x, Thermo Fisher Scientific, catalog number: 35050038), culture one supplement (100x, Thermo Fisher Scientific, catalog number: A3320201), Ascorbic acid 2-phosphate-sesquimagnesium salt hydrate (200 mM, Sigma Aldrich, catalog number: A8960) and antibiotic–antimycotic (100x, Thermo Fisher Scientific, catalog number: 15240096). The culture medium was changed every 3 days, for 15 to 28 days. Neurons generated were collected for RNA analysis and immunostaining assays.

### Quantitative real time PCR (qPCR)

qPCR assays based on SYBR-Green was performed for several markers, used to characterize NSCs and neurons lineages (Table 1). Briefly, total RNAs were extracted from specific cell culture, and purified using Macherey–Nagel NucleoSpin® RNA II kit (Macherey–Nagel, catalog number: 740955.250), according to the manufacturer’s protocol. A TURBO-DNase (Thermo Fisher Scientific, catalog number: AM1907) treatment was performed on RNA extracted, to ensure the elimination of residual genomic DNA. RNAs were retro-transcribed by ImProm-II™ Reverse Transcription System (Promega, catalog number: A3800), following the specific protocol. Assays have been carried out using iTaq™ Universal SYBR® Green Supermix, (Bio-Rad, catalog number: 1725121), 10 ng of total cDNA and 200 mM of each primer. The annealing temperature has been set at 60 °C.

**Table 1:** Primers set used for characterization of generated neuronal cell lines (qPCR assays based on SYBR-Green)

Gene	Forward 5′ −3′	Reverse 5′−3′
ACTIN	CGCCGCCAGCTCACCATG	CACGATGGAGGGGAAGACGG
OCT4	CCTCACTTCACTGCACTGTA	CAGGTTTTCTTTCCCTAGCT
SOX2	CCCAGCAGACTTCACATGT	CCTCCCATTTCCCTCGTTTT
NESTIN	GCGTTGGAACAGAGGTTGGA	TGGGAGCAAAGATCCAAGAC
SOX1	CCTCCGTCCATCCTCTG	AAAGCATCAAACAACCTCAAG
PAX6	CTGAAGCGGAAGCTGCAAAG	TTGCTGGCCTGTCTTCTCTG
MAP2	GACTGCAGCTCTGCCTTTAG	AAGTAAATCTTCCTCCACTGTGAC
DCX	TATGCGCCGAAGCAAGTCTC	TACAGGTCCTTGTGCTTCCG
TUBB3	GGCCAAGTTCTGGGAAGTCAT	CTCGAGGCACGTACTTGTGA
GFAP	CTGCGGCTCGATCAACTC	TCCAGCGACTCAATCTTCCTC
GAD	GTCGAGGACTCTGGACAGTA	GGAAGCAGATCTCTAGCAAA
CHAT	ACTGGGTGTCTGAGTACTGG	TTGGAAGCCATTTTGACTAT
TH	TCATCACCTGGTCACCAAGTT	GGTCGCCGTGCCTGTACT

qPCR for gene expression in control and patient cell lines was performed using TaqMan Gene Expression Master mix (Applied Biosystems catalog number: 4369016), 10 ng of total cDNA, and using Taqman probe assays: (HS00389316-m1, TPPP gene; HS01549381-m1 SEMA5 A gene; HS00181643-m1 CTNND2 gene; HS00972656-m1, TERT gene; HS02758991-g1, GAPDH gene) (Thermo Fisher Scientific). All samples were analyzed in triplicates.

qPCR assays were performed on CFX96 C1000 Touch™ Real-Time PCR Detection System (Bio-Rad) and analyzed with Bio-rad CFX manager software v.3.1. (Bio-Rad). The relative quantification was calculated using the 2^−ΔΔCT^ method.

### Immunofluorescence assay

Immunofluorescence assays was performed on adherent cells cultured on glass slides (Steroglass, catalog number: KITY025653) inserted in 24 well, to verify the expression of specific markers of NSCs and neurons. Initial washing was done with 1X phosphate-buffered saline (PBS) before fixing for 15 min with Reagent A (Fix & Perm-Reagent A, SIC, catalog number: GAS-002 A-1). After fixing, the cells were either stored in 1X PBS at 4 °C or stained immediately. Permeabilisation was performed with Reagent B (Fix & Perm-Reagent B, SIC, catalog number: GAS-002B-1) for 15 min. Then a blocking solution (iBindTM 5X Buffer-Invitrogen, catalog number: SLF1020) has been applied for 45 min. After blocking, cells were incubated with primary antibodies (Table 2) diluted in permeabilization Reagent B for 3 h at room temperature. After incubation with primary antibodies, cells were washed three times with 1% BSA in 1X PBS, and incubated light-protected with secondary antibodies (Table 2) diluted in permeabilization Reagent B for 1 h at room temperature. Following incubation, cells were washed twice with 1X PBS and incubated in 4',6-diamidino-2-phenylindole (DAPI, Thermo Fisher Scientific, D1306) diluted in 1X PBS for 5 min at room temperature. Finally, the glass slides were mounted with Glycerol Gelatin (Sigma Aldrich, catalog number: GG1) and stored at 4 °C in the dark overnight. Cells have been observed under a fluorescence microscope, Olympus IX70 inverted microscope and images analyzed with the Image-Pro Plus software v7.0 (Media Cybernetics).

**Table 2:** Antibodies used for cell characterization with immunofluorescence assay

Typology	Name antibody	Catalog number	Dilution	Host
Primary Ab	NESTIN	Thermo Fisher Scientific, A24345	1:100	mouse
Primary Ab	MAP2	Invitrogen, 13–1500	1:300	mouse
Primary Ab	TH	Sigma Aldrich, AB152	1:200 (IF) 1:1000(WB)	Rabbit
Primary Ab	DAT	GeneTex, GTX133152	1:1000	Rabbit
Primary Ab	GAPDH	Santa Cruz, sc-365062	1:1000	Mouse
Secondary Ab	Goat anti rabbit IgG (H + L) Alexa Fluor 568	Thermo Fisher Scientific, A-11011	1:250	Rabbit
Secondary Ab	Goat anti mouse IgG (H + L) Alexa Fluor 568	Thermo Fisher Scientific, A-11004	1:200	Mouse
Secondary Ab	Goat anti rabbit IgG (H + L) Alexa Fluor 488	Thermo Fisher Scientific, A-11008	1:200	Rabbit
Conjugated Ab	Alexa FluorTM 488 Phalloidin	Invitrogen, A12379	1:500	
Secondary Ab	Goat anti-Rabbit IgG (H + L) Secondary Antibody, HRP	Invitrogen, 31,460	1:8000	Goat
Secondary Ab	Goat anti-Mouse IgG (H + L) Secondary Antibody, HRP	Invitrogen, 31,430	1:8000	Goat

### Western blot

Cells were lysed with RIPA bufer (Merck Millipore) supplemented with Halt™ protease and phosphatase inhibitor cocktail and EDTA (Thermo Fisher Scientific). Protein concentration was determined by Bradford assay (BioRad). Twenty micrograms of protein were run on NuPAGE™ 4–12% Bis–Tris polyacrylamide gels (Invitrogen, Thermo Fisher Scientifc) in denaturing and reducing conditions. Proteins were then transferred onto PVDF membranes (GE Healthcare, Chicago, IL). Membranes were blocked with 5% non-fat milk in TBST and incubated with primary antibodies overnight at 4 °C. After blocking, membranes were incubated with primary antibodies (Table 2) diluted 1:1000 in in 1% milk-TBST, for overnight at 4 °C. After washing, membranes were incubated with secondary horseradish peroxidase (HRP)-conjugated antibody (Table 2) diluted in 1% milk-TBST, for 1 h at room temperature. After washing, the signal was detected with Westar ηC Ultra chemiluminescent substrate (Cyanagen, Bologna, Italy) on iBright CL1000 Imaging System (Invitrogen, Thermo Fisher Scientifc), and band densitometry was analyzed on iBright analysis software (Thermo Fisher Scientifc). Traget signal intensity was normalized to *housekeeping* GAPDH; HD sample was used as reference.

## Results

### Derivation of expandable population of neural stem cells

**Fig. 1:**
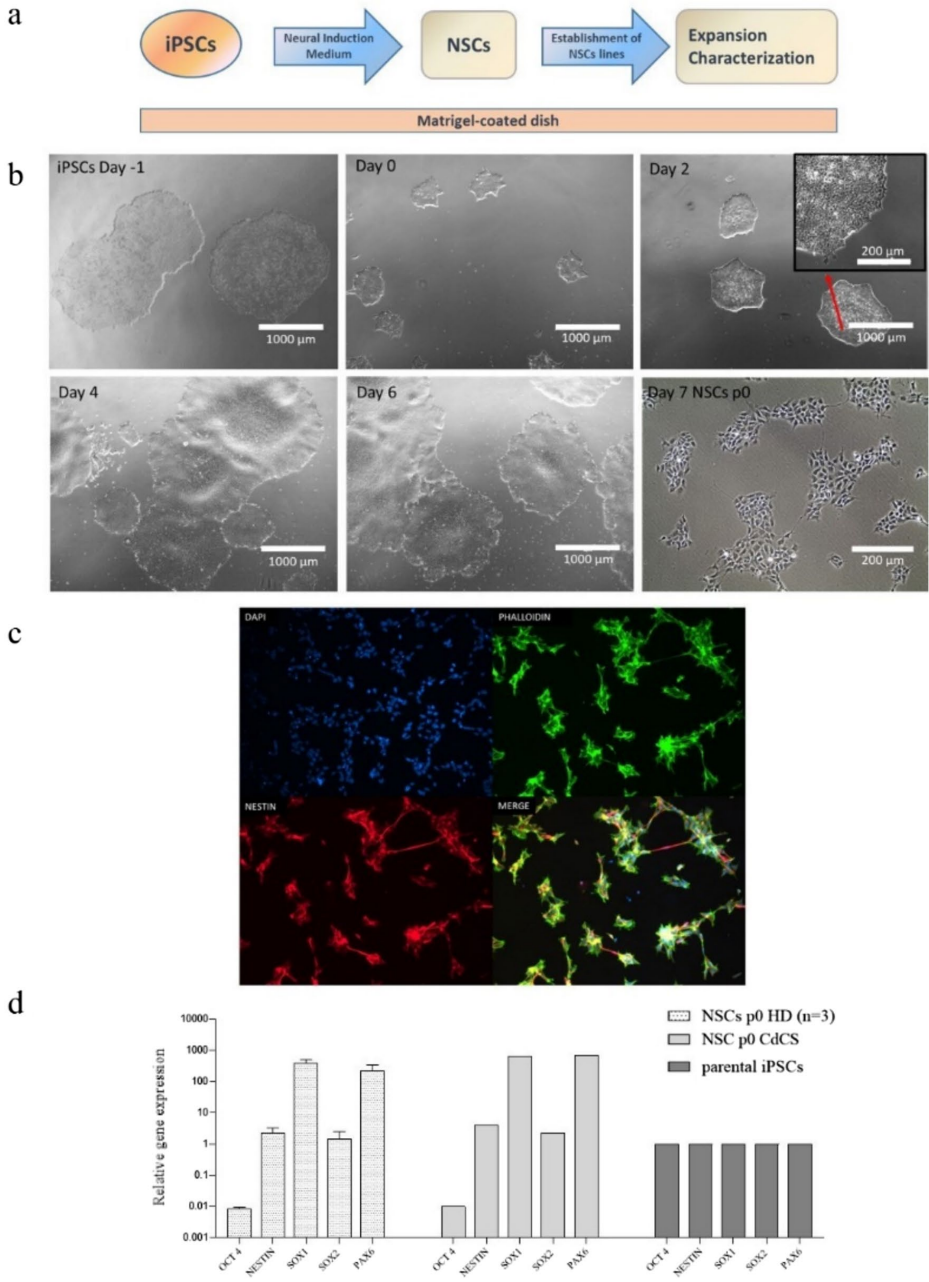
CdC-NSCs differentiation **a** Experimental workflow. **b** NSCs differentiation: iPSCs colonies were transferred to Matrigel-coated plates and culture in xeno- and serum-free Gibco® PSC Neural Induction Medium (ThermoFisher Scientific) for 7 days. At day 7 of neural induction, primitive NSCs were enzymatically dissociated and plated on Matrigel-coated dishes for expansion and cryopreservation. **c** Immunofluorescence assay performed on NSCs at p0 with antibodies against NSCs markers Nestin (red), and against Phalloidin (green), Magnification 20X. **d** q-PCR assay for *NESTIN, SOX1, SOX2, OCT4* and *PAX6* expression in NSCs p0 obtained. Data were normalized on *ACTIN* and calculated in relation to parental iPSCs line (black bars). NSCs show an increased gene expression of neural markers *NESTIN, SOX1, SOX2,* and *PAX6*. A reduced gene expression of pluripotency marker *OCT4* indicates the absence of residual iPSCs not induced. The standard deviation was calculated from gene expression of three distinct HD-derived iPSCs clones (n = 3)

The neural induction workflow is described in Fig. 1a. The iPSCs transition to NSCs phenotypes is shown in Fig. 1b. It is noteworthy that during the neural induction phase a change in iPSCs morphology was observed. At the initial stages of neural induction, cells expanded and displayed the typical iPSCs colonies shape: small round cells, and uniform surface with well defined edge. As the duration of the culture progressed to day 2–4 cells exhibited a rounder morphology with a very expanded and dense nucleus. By days 6/7 cells propagated and grew in a distinct uniform monolayer with some elongated cells emerging from the colonies.

The acquisition of the correct neuronal phenotype was first assessed by qualitative immuno-fluorescence staining on NSCs at passage 0 (p0) showing the presence of the NSCs markers, NESTIN, and the cytoskeletal marker Phalloidin, Fig. 1c.

Finally, to deepen the NSCs characterization, gene expression analysis of NSCs markers *NESTIN, SOX1, SOX2, PAX6*, and the pluripotent marker *OCT4* was performed. The analysis in SYBR Green qPCR was conducted on RNAs collected at the end of the induction process (day 7, NSCs p0) comparing the newly generated NSCs with the parental iPSCs, set as calibrator. In Fig. 1d the activation of NSCs genes expression, associated to a strong reduction of iPSCs marker *OCT4* is shown. The strong reduction of *OCT4* expression prove the absence of residual untransformed iPSC colonies. CdC-derived NSCs display a similar expression pattern to the HD-derived NSCs, with prominent increase of *SOX1*, *PAX6*, and an expected moderate increment of *SOX2* and *NESTIN.*

These results suggest that cells derived from iPSCs through neural induction process possess the NSC phenotype and, exploiting their multipotency ability, can be used to generate a population of neurons in vitro*.*

Cytogenetic analysis revealed no acquired chromosomal abnormalities, demonstrating maintained karyotypic integrity in the neural stem cell population. The standard karyotype confirmed chromosomal stability and, consequently, the 5p deletion that had previously characterized the PBMC and UNI BSi004-A hiPSCs lines derived from a patient with CdC syndrome [37]

**Fig. 2:**
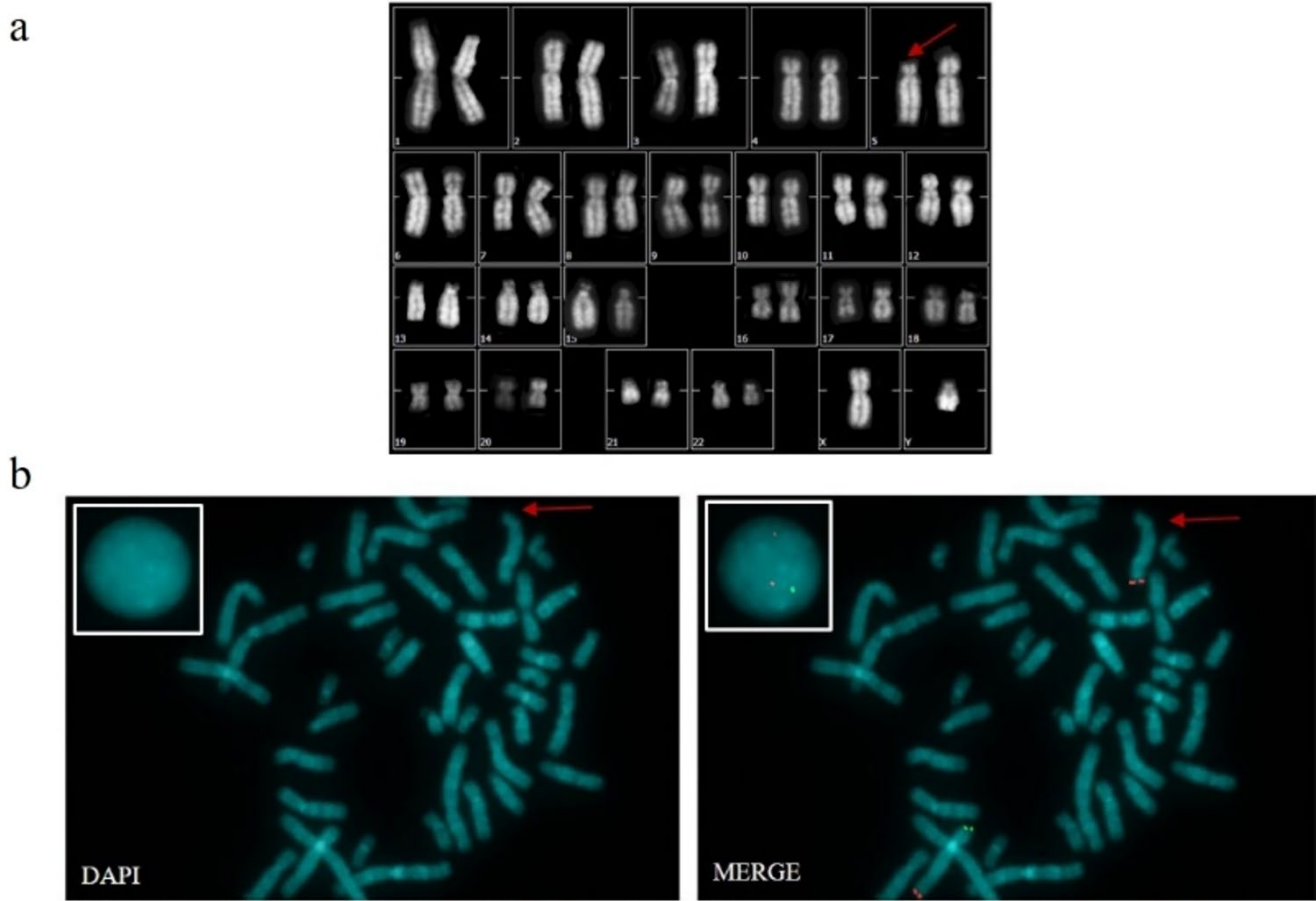
Karyotype and FISH **a** Karyotype, the arrow indicates chromosome 5 with the deletion of the short arm. **b** Metaphase stained with DAPI to counterstain the chromosomes. In the square bordered in white, an interphase is inserted; Merge in the metaphase plate and in the interphase within the white-bordered square, both control signals for the 5qter region appear in red, while only one signal for the candidate 5p (CdCCR) region is visible in green, highlighting the deletion of the short arm of chromosome 5. The red arrow indicates the deleted chromosome 5, which lacks the green signals. Magnification 100X

FISH confirmed a constitutional deletion of the short arm (5p15) at the CdCCR locus, characterized by absent hybridization signals in this critical region (Fig. 2b). This integrated approach validates both global genomic stability and the presence of the 5p deletion, providing essential quality control data for downstream experimental applications.

### CdC-NSCs can be differentiated to neuronal lineages

**Fig. 3:**
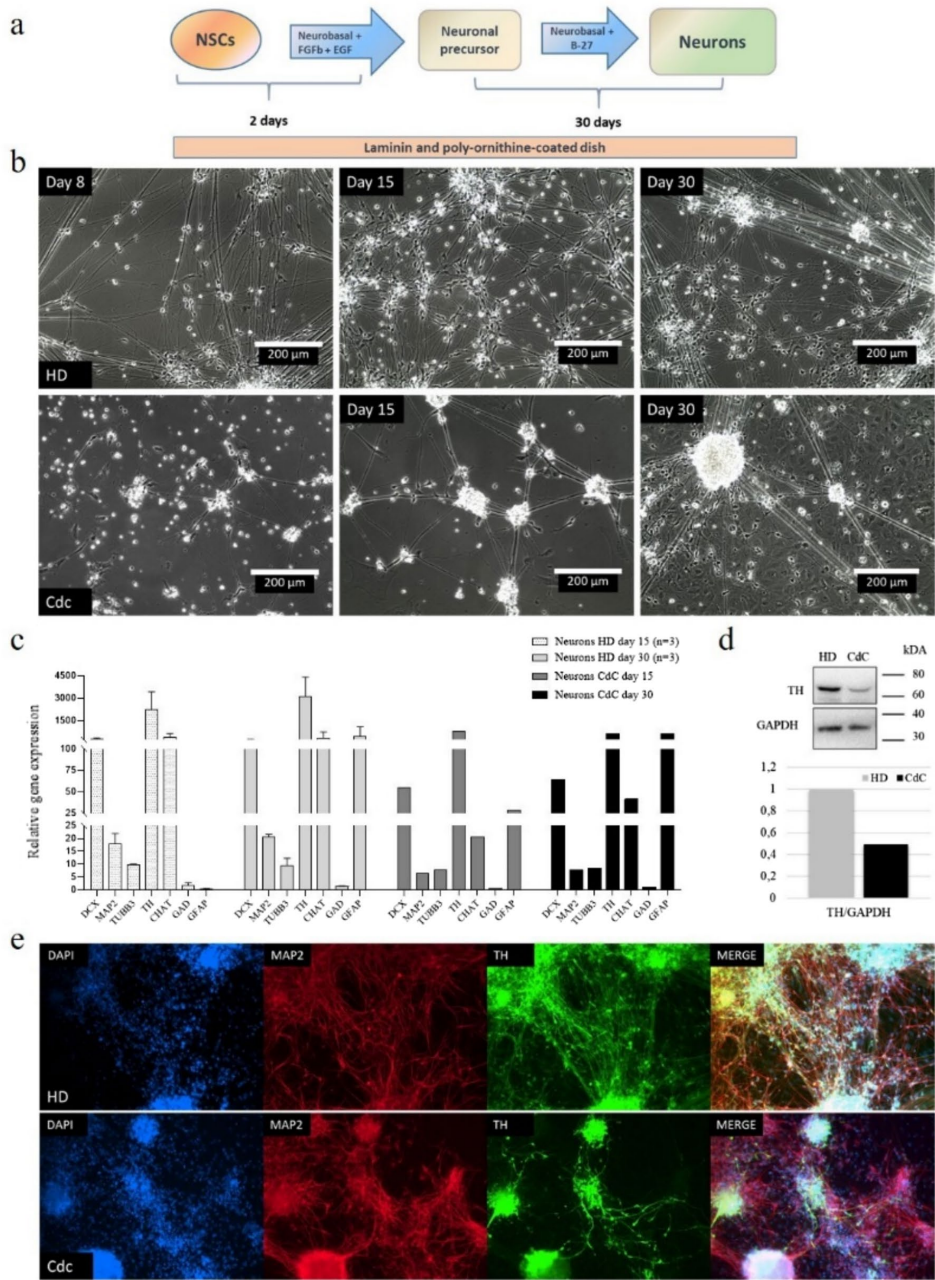
CdC-derived neurons **a** Experimental workflow. **b** Morphologic modifications occurred during differentiation into neurons. Scale bar of 200 μm. **c** qPCR analysis for *DCX, MAP2, TUBB3, TH, GAD, CHAT*, and *GFAP* gene expression in a CdC- and HD-derived neurons at day 15 and day 30 of differentiation. Data were normalized on *ACTIN* and calculated in relation to parental NSCs line at passage 0. The standard deviation was calculated from gene expression of three distinct HD-derived iPSCs clones (n = 3). **d** Western blot analysis and band densitometry graph of TH expression in CdC- and HD-derived neurons at day 30 of differentiation. TH signal was normalized to GAPDH. **e** Immunofluorescence images of healthy donor (HD) and CdCS derived neurons stained with antibodies against the neuronal marker MAP2 in red and the dopaminergic marker TH in green; nuclei are stained with DAPI in blue, Magnification 10X

Expanded HD and CdC-NSCs at passage 10 were differentiated in neurons (Fig. 3a). The differentiation process led to the growth of a heterogeneous neuronal population after 30 days in culture. The morphologic modifications occurred during neuronal differentiation are reported in Fig. 3b. At day 8, cells under differention stimuli spread on the plate and assume a more elongated shape in comparison to their parental NSCs that tend to grow clustered. Over several days the cells were in over confluence and began to form aggregates that reminds the neuronal rosettes [42].

To validate the neuronal phenotype acquisition of derived neurons*,* we analyzed by qPCR a gene expression panel in RNAs collected at day 15 and 30 of neuronal differentiation. The qPCR assays have included neuronal cytoskeletal markers as doublecortin (*DCX*), microtubule associated protein 2 (*MAP2*) and β III tubulin (*TUBB3*), and others specific neuronal subpopulations markers as the glutamic acid decarboxylase (*GAD*) expressed in gabaergic neurons, the choline acetyltransferase (*CHAT*) expressed in cholinergic neurons, the tyrosine hydroxylase (TH) expressed in dopaminergic neurons and the glial fibrillary acidic protein (*GFAP*) encoded by the glial cells. Data were normalized on *ACTIN* and calculated in relation to parental NSCs line at passage 0. The graph shown in Fig. 3c reports the increase of all the neuronal markers in both HD and CdC NSCs-derived neurons apart from *MAP2* and *TUBB3*, whose increases are less appreciable following the already consolidated expression in parental NSCs. Both HD and CdC neurons show as predominant subcellular population the dopaminergic one, represented by the strong induction of *TH*, followed by the cholinergic (*CHAT*) and finally the gabaergic (*GAD*). Moreover, all neuronal cultures at 15 days of differentiation showed a negligible level of *GFAP* that strongly increased after 30 days. Finally, these results suggest that both HD and CdC-derived NSCs can generate a heterogenous population of neurons endowed of the typical neuronal marker of maturity such *MAP2* and *TH*. Comparing the HD and CdC performance, is notable that CdC-derived neurons at day 30 have a strong reduction of the *TH* and *CHAT* positive population and a slight significant increase in the *GFAP* positive cells. The huge decrease of *TH* was confirmed also at protein level by western blot analysis in neurons at day 30 of differentiation. TH expression in CdC-derived neurons is about half in comparison to HD-derived neurons (Fig. 3d). Finally, we analyzed by immunofluorescence the protein expression of the neuro-cytoskeletal MAP2 marker, and the dopaminergic target TH. As shown in Fig. 2d, MAP2 is well expressed in both the control and the patient cells but with a different distribution: in the control, the differentiation has led to a well-organized and distributed neuronal network, whereas in the patient it appears minor and less structured. Moreover, the reduced expression of TH in the CdC neurons compared to HD, observed before by western blot was confirmed also by immunofluorescence.

### Quantitative real time (qRT)-PCR deleted genes

We carried out the expression study of *TERT, CTNND2, SEMA5 A* and *TPPP,* genes deleted in the heterozygous state, comparing their expression level in the iPSCs, NSCs and Neurons lines generated in vitro from HD and CdC cells.

**Fig. 4:**
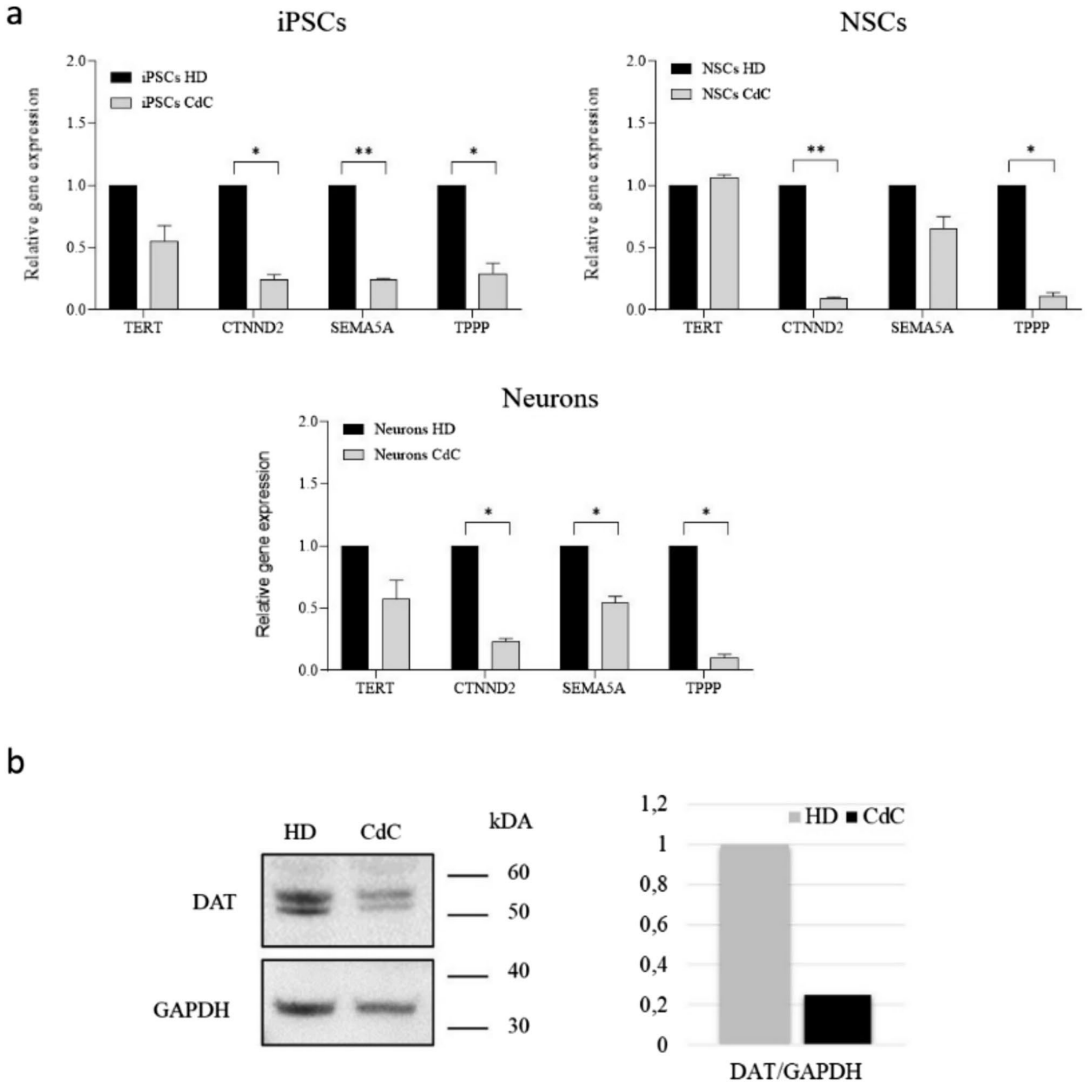
Expression deleted genes **a** Quantitative Real Time PCR (qPCR) analysis for *TERT, CTNND2, SEMA5 A* and *TPPP* gene expression in iPSCs. **b** in NSCs. **c** Neurons. Data were normalized on *GAPDH* and plotted in relation to values of HD as calibrator (black bars). The bars indicate the standard deviation calculated from three distinct experimental replicates (n = 3). Statistical significance was determined using an unpaired Student’s t test for each gene. The asterisks indicate significant differences between CdC-derived cells and healthy donor- derived cells (*p < 0.05, **p < 0.001). **b** Western blot analysis and band densitometry graph of DAT expression in CdC- and HD-derived neurons at day 30 of differentiation. DAT signal was normalized to GAPDH

In CdC- iPSCs, *TERT* gene expression is half as compared to the HD, *CDNND2* value five times lower, *SEMA5 A* gene expression is four times lower and *TPPP* about three times lower (Fig. 4a).

In CdC- NSCs, *CDNND2* expression value is ten times less, *SEMA5 A* is about a half and TPPP about ten times less than HD-derived NSCs while *TERT* expression is similar in the two populations (Fig. 4b).

In CdC-neurons, *TERT* gene expression is half as compared to HD, *CDNND2* value is four times lower, *SEMA5 A* gene expression is half and *TPPP* about ten times less (Fig. 4c).

The strong reduction of *CDNND2, SEMA5 A,* and *TPPP* expression showed in CdC-derived iPSCs, NSCs, and neurons is statistically significant. Finally, the protein expression of DAT (Dopamine transporter) encoded by *SLC6 A3*, gene deleted in the heterozygous state in the patient’s cells was assessed in neurons at day 30 of differentiation. As shown in Fig. 4b, the DAT expression in CdC-derived neurons is about 80% less in comparison to HD-derived neurons.

## Discussion

CdC syndrome scientific research is focused only on clinical and instrumental studies of affected patients, well defining the phenotype. As far as CdC syndrome is concerned, to date there are no animal models to study this rare chromosome disease. Our research group developed the first iPSCs model from PBMCs of a patient affected by CdC Syndrome [37]. Starting from these cells, we established and characterized a novel NSCs line. The stabilization, phenotypic matching of these cells and their multipotency ability allowed us to further differentiate them into heterogeneous neuronal cells to highlight, if possible, which neuronal subpopulation may be most involved in the pathologic process of the syndrome. The choice of this target is dictated by the severe delay and neuro-motor development present in 100% of CdC patients caused by the characteristic deletion of the short arm of one chromosome 5, which translates into the loss of a copy of genes mapped in the 5 short arm. Our research visualizes a CdC-induced neuronal population, at 30 days of in vitro growth. The overview showed different patterns between the CdC and HD neuronal subpopulations, confirmed by the relative expressions. Comparing the gene expression panel for *DCX, MAP2, TUBB3, TH, GAD, CHAT*, and *GFAP* for the different types of neurons, the dopaminergic one is the predominant neuronal population both in HD and CdC-cells, followed by the cholinergic and gabaergic.

In CdC- neuronal line there is a general reduction in all the markers at both 15 and 30 days of growth, expecially for *TH* and *CHAT* genes, compared to the HD levels. These results might suggest that there may be both a low number but also a reduced functionality of subpopulations, probably related to deletion of above-mentioned genes. The reduced *CHAT* expression is reported in Alzeimer patients where a 60–95% decrease in CHAT activity has been noted [46, 47], index for down-functionality of cholinergic neurons, and, as we observe the same phenomenon, extensive studies are needed to define if the CHAT-positive population in CdC-induced neurons are hypofunctional. GABAergic neurons type is reduced in the CdC neuronal population and the obtained results of immunofluorescence on CdC TH positive neurons showed a very uneven appearance. GFAP gene expression is slightly increased in CdC. GFAP is a marker expressed by a heterogeneous population of the glia cells that have different roles in in the adult mammalian brain function such as myelination, synaptic function, modulation of homeostatic functions, nerve signal propagation and responses to neural injury [48–51]. GFAP is adopted as a marker of astrocytes in the central nervous system (CNS). GFAP levels increase as a nearly universal response to injury and disease, when astrocytes enter a state of “reactive gliosis” [52]

It will be interesting to differentiate only the astrocytic population and study its morphology, as well as do more in-depth studies on the microglia population because its activation or dysfunction can be associated with neuroinflammation, brain and energy metabolism as reported by Cistaro in a group of CdC patients [9].

The results of our study regarding gene expression of selected genes might help us to make hypotheses to understand and correlate the data obtained from neuronal differentiation.

At first, the very low relative expression we obtained suggests a downregulation of *TPPP/p25* gene. More recently, *TPPP/p25* was shown to be specifically expressed in myelinating oligodendrocytes important for oligodendrocyte differentiation and microtubule assembly in vitro [53, 54]. In the CNS, myelination is carried by oligodendrocytes derivated from oligodendrocyte progenitor cells (OPCs), which hold the capacity to proliferate, migrate, and differentiate into myelinating oligodendrocytes. It has been shown that at substoichiometric concentrations, *TPPP/p25* promotes the polymerization of tubulin into aberrant forms, such as double-walled tubules and aggregates [55]. Our results seem indicate that very low expression might prevent oligodendrocyte differentiation.

*TERT* gene, in iPSCs and neuronal populations, is expressed respecting the hemizygosity, while in NSCs it is the same as the HD, perhaps highlighting an up-expression. Saretzki [27, 56] reports attributing an extra-telomerase role to the TERT protein, associating over-expression with a possible protective role, even if not entirely clear, against potential toxigenic proteins involved in neurodegeneration, already observed in post-mortem studies on a CdC patient [10]. There is interest in the role of telomerase protein for brain function, such as cognitive ability, aging, brain injury, and memory [57–61].

For *SEMA5 A* gene, the expression value found in all three types of cell lines was half of the HD exactly as we expected. Regarding *CTNND2* gene, the low-expression value found in all three cell line types was surprising and unexpected. ð-catenin is a critical regulator of spine architecture in developing and mature neurons. In mouse model loss of ð -catenin leads to a reduction in the head width and length of spines [62]. However, our results are insufficient to understand how much the absence of an allele influences the development of dendritic spines and therefore more in-depth studies performed on different types of neuronal cells, will be necessary.

In conclusion, we successfully established and characterized new CdC NSCs from CdC-iPSCs, which allowed us to perform subsequent differentiation in a heterogeneous population of neurons. The differences shown between control and syndrome suggested to us that in future, it will be necessary to differentiate individual neuronal subtypes such as dopaminergic, gabaergic, oligodentrocytic, and astrocytic for their possible prominent role in the pathology of the CdC patient, starting from aur CdC NSCs. After confirming a reduction in the dopaminergic population positive for TH staining in CdC-derived neurons, to deepen the validation of the in vitro disease model, we also reported and discussed, for the first time, the gene expression value of *CTNND2, SEMA5 A, TPPP, TERT* genes, present in hemizygosity, in the three types of cell lines. Moreover, we examined the protein expression of DAT, the dopamine transporter, encoded by the *SLC6 A3* gene, deleted in heterozygosity in CdC. We observe a strong reduction of DAT expression in CdC-derived neurons in comparison to HD-derived neurons. In healthy dopaminergic neurons, there is often a positive correlation between TH and DAT expression. This is because dopaminergic neurons not only produce dopamine (via TH) but also rely on the transporter (DAT) to recycle dopamine after its release into the synapse, ensuring proper dopamine signaling. Reduced DAT expression could contribute to lower dopamine production, because of the reduced recycle of the dopamine [63, 64].

The neural stem cells and neurons obtained will be a valuable and very important aid for further in vitro functional research on CdC syndrome, advancing the understanding of the pathologic mechanisms underlying the disease and contributing to the development of possible innovative treatments.

## Data Availability

Data supporting findings reported in this study are available from the corresponding author upon reasonable request.
